# The Additional Value of Ultrafast DCE-MRI to DWI-MRI and 18F-FDG-PET to Detect Occult Primary Head and Neck Squamous Cell Carcinoma

**DOI:** 10.3390/cancers12102826

**Published:** 2020-09-30

**Authors:** Roland M. Martens, Ruud van der Stappen, Thomas Koopman, Daniel P. Noij, Emile F. Comans, Gerben J. Zwezerijnen, Marije R. Vergeer, C. René Leemans, Remco de Bree, Ronald Boellaard, Jonas A. Castelijns, Pim de Graaf

**Affiliations:** 1 Department of Radiology and Nuclear Medicine, Amsterdam UMC, Vrije Universiteit Amsterdam, Cancer Center Amsterdam, De Boelelaan 1117, 1081 HV Amsterdam, The Netherlands; r.vanderstappen@amsterdamumc.nl (R.v.d.S.); t.koopman@amsterdamumc.nl (T.K.); daannoij@gmail.com (D.P.N.); e.comans@haaglandenmc.nl (E.F.C.); g.zwezerijnen@amsterdamumc.nl (G.J.Z.); r.boellaard@amsterdamumc.nl (R.B.); j.castelijns@nki.nl (J.A.C.); p.degraaf@amsterdamumc.nl (P.d.G.); 2 Department of Radiation Oncology, Amsterdam UMC, Vrije Universiteit Amsterdam, Cancer Center Amsterdam, De Boelelaan 1117, 1081 HV Amsterdam, The Netherlands; mr.vergeer@amsterdamumc.nl; 3 Department of Otolaryngology—Head and Neck Surgery, Amsterdam UMC, Vrije Universiteit Amsterdam, Cancer Center Amsterdam, De Boelelaan 1117, 1081 HV Amsterdam, The Netherlands; cr.leemans@amsterdamumc.nl; 4 Department of Head and Neck Surgical Oncology, University Medical Center Utrecht, Heidelberglaan 100, 3584 CX Utrecht, The Netherlands; r.debree@umcutrecht.nl

**Keywords:** unknown primary, ultrafast DCE, DWI, PET, MRI, head and neck neoplasms

## Abstract

**Simple Summary:**

Patients with cervical lymph node metastasis from squamous cell carcinoma undergo extensive irradiation or surgery of the head and neck with higher treatment morbidity, recurrence rate and lower overall survival than patients with overt primary tumor. In order to enhance treatment efficiency and morbidity reduction, the primary tumor detection accuracy was evaluated by using Ultrafast-Dynamic Contrast-Enhancement (DCE-)MRI in addition to Diffusion-Weighted (DW-)MRI and ^18^F-FDG-PET/CT imaging. Ultrafast-DCE, with a temporal resolution of 4 s, enabled capturing lesions with increased neoangiogenesis or perfusion compared to normal tissue. The use of Ultra-fast DCE resulted in higher confidence for suspicious locations and high observer agreement. Ultrafast-DCE showed potential to improve detection of unknown primary tumors in addition to DWI and ^18^F-FDG-PET/CT in patients with cervical squamous cell carcinoma lymph node metastasis. The combined use of ultrafast-DCE, DWI and ^18^F-FDG-PET/CT yielded highest sensitivity.

**Abstract:**

To evaluate diagnostic accuracy of qualitative analysis and interobserver agreement of single ultrafast-DCE, DWI or ^18^F-FDG-PET and the combination of modalities for the detection of unknown primary tumor (UPT) in patients presenting with cervical lymph node metastasis from squamous cell carcinoma (SCC). Between 2014–2019, patients with histologically proven cervical lymph node metastasis of UPT SCC were prospectively included and underwent DWI, ultrafast-DCE, and ^18^F-FDG-PET/CT. Qualitative assessment was performed by two observers per modality. Interobserver agreement was calculated using the proportion specific agreement. Diagnostic accuracy of combined use of DWI, ultrafast-DCE and ^18^F-FDG-PET/CT was assessed. Twenty-nine patients were included (20 males. [68%], median age 60 years). Nine (31%) primary tumors remained occult. Ultrafast-DCE added reader confidence for suspicious locations (one additional true positive (5%), 2 decisive true malignant (10%). The per-location analysis showed highest specific positive agreement for ultrafast-DCE (77.6%). The per-location rating showed highest sensitivity (95%, 95%CI = 75.1–99.9, YI = 0.814) when either one of all modalities was scored positive, and 97.4% (95%CI = 93.5–99.3, YI = 0.774) specificity when co-detected on all. The per-patient analysis showed highest sensitivity (100%) for ^18^F-FDG-PET/CT (YI = 0.222) and either DWI or PET (YI = 0.111). Despite highest trends, no significant differences were found. The per-patient analysis showed highest specific positive agreement when co-detected on all modalities (55.6%, 95%CI = 21.2–86.3, YI = 0.456). Ultrafast-DCE showed potential to improve detection of unknown primary tumors in addition to DWI and ^18^F-FDG-PET/CT in patients with cervical squamous cell carcinoma lymph node metastasis. The combined use of ultrafast-DCE, DWI and ^18^F-FDG-PET/CT yielded highest sensitivity.

## 1. Introduction

Head and neck squamous cell carcinoma (HNSCC) presents with cervical lymph node metastasis of an unknown primary tumor (UPT) in up to 9% of patients [1,2,3]. Location of primary HNSCC remains occult in 60–80% of patients after extensive diagnostic workup including physical examination and assessment under anesthesia [4]. These UPT patients undergo (chemo)radiotherapy, generally including the whole mucosal area where potentially the occult primary tumor may be hidden, with or without neck dissection [5]. This (too) extensive irradiation results in higher treatment morbidity, recurrence rate and lower overall survival than patients with overt HNSCC [4,5,6]. Therefore, detection of the primary tumor location is vital to allow for more tumor focused radiotherapy or surgery [5,7].

Functional imaging techniques, such as DWI and ^18^F-FDG-PET)/CT, can capture unique tumoral characteristics and are used nowadays to improve tumor detection. DWI captures tissue cellularity (i.e., restricted diffusion in HNSCC), which is quantified as lower ADC values compared to benign tissue [8]. However, in tonsillar carcinoma higher ADC values are reported compared with normal (lymphoid) tonsils, which can therefore result in a diminished detection rate, especially in small primary tumors [1,9,10].

Currently, ^18^F-FDG-PET is the standard imaging modality to detect UPT by detecting tumor metabolism, i.e., high ^18^F-FDG uptake in HNSCC [3,11,12]. However, due to limited anatomical detail and low imaging resolution, detection of small (<5 mm) lesions is limited [13]. Moreover, also physiological uptake appears in normal anatomical structures causing false-positive results, e.g., high ^18^F-FDG-PET/CT-tracer uptake in tonsillar tissue [1,14], due to hypertrophic lymphoid tissue, tonsillitis [1,13] or tissue manipulation (e.g., diagnostic endoscopy) [3].

Ultrafast-DCE with a high temporal resolution (i.e., <10 s per stack of images) captures details of the first contrast uptake kinetics and increased the diagnostic performance for tumor detection in prostate [15] and breast cancer [16,17]. The principle of discrimination between benign and malignant lesions is based on the earlier time of arrival of the contrast in malignancies with increased neoangiogenesis or perfusion compared to normal tissue. Previous studies described significant correlations between DCE-parameters and histological parameters with a temporal resolution of 6 s [18], described potential for semi- and quantitative identification of posttreatment recurrence with a temporal resolution of 2–4 s [19], and found improved differentiation of malignant tumors from post radiation changes using temporal resolution of 2.6 s [20], but the diagnostic accuracy using ultra-fast-DCE was not described. We hypothesized that early enhancement after contrast media injection (i.e., ultrafast-DCE with temporal resolution at 4 s) might also allow for a more accurate detection of primary tumor location of unknown primaries in the head and neck area.

The purpose of this study is to evaluate diagnostic accuracy of qualitative analysis and interobserver agreement of single ultrafast-DCE, DWI or ^18^F-FDG-PET and the combination of modalities for the detection of UPT in patients presenting with cervical squamous cell carcinoma (SCC) lymph node metastasis.

## 2. Results

The study population consisted of 29 patients (20 males, 69.0%), median age 60 years, range 45–76 years) (Table 1, Figure S1). The performance of imaging and pathology resulted in 20 discovered primary tumor lesions (69%) and in 9 patients (31%) primary tumor remained occult at final diagnosis (Table S1). Forty locations out of the total of 174 locations (23%) were scored positively for malignancy, whereas 20 of these locations (50%) were histopathologically confirmed by the examination under general anesthesia (EUA).

### 2.1. Diagnostic Performance of Single DWI, DCE or ^18^F-FDG-PET/CT Analysis

The final diagnosis for each patient and the consensus score for each modality (Table 2), showed that all modalities were in good agreement. DWI and DCE showed both 9 false-positive locations (5.1%) and ^18^F-FDG-PET/CT 14 false-positive locations (8%).

The per-location analysis (Table 3 I) resulted in a sensitivity of 80% (95%CI = 56.3–94.3, YI = 0.735) for DWI, 95% (95%CI = 75.1–99.9, YI = 0.829) for ultrafast-DCE, and 90% (95%CI = 68.3–98.8, YI = 0.796) for ^18^F-FDG-PET/CT. A per-location specificity of 93.5% (95%CI = 88.4–96.8) was found for DWI, 92.9% (95%CI = 87.6–96.4) for ultrafast-DCE, and 89.6% (95%CI = 83.7–94.0) for ^18^F-FDG-PET/CT (Table 3 I).

The per-patient analysis (Table 4 I) resulted in sensitivity of 95% (95%CI = 75.1–99.9, YI = 0.117) for DWI, 90% (95%CI = 75.1–99.9, YI = 0.233) for ultrafast-DCE, and 100% (95%CI = 83.2–100, YI = 0.222) for ^18^F-FDG-PET/CT. A per-patient 33.3% specificity was found for ultrafast-DCE, whereas other modalities showed 22.2% specificity (Table 4; I). There were no significant differences in sensitivity or specificity between each modality (not tabulated).

### 2.2. Diagnostic Performance of the Combined Use of DWI, DCE and ^18^F-FDG-PET/CT

In the per-location analysis, the combined use of modalities with an independent detection (positive read) in either DWI, ultrafast-DCE or ^18^F-FDG-PET/CT, resulted in the highest sensitivity of 95% (95%CI = 75.1–99.1%) (Table 3; II and III). This was non-significantly higher than either DWI or ultrafast-DCE (both sensitivity = 90% (95%CI = 68.3–98.8%, *p* = 1.0)) and higher than either DWI or PET (sensitivity = 90% (95%CI = 68.3–98.8%, *p* = 1.0). In this analysis, the specificity was 90.3% (95%CI = 84.4–94.5) for either DWI or ultrafast-DCE. This was non-significantly higher than for either PET or DWI (specificity = 87%, 95%CI = 80.7–91.9, *p* = 0.125), and significantly higher than either DWI, DCE or PET (specificity = 86.4%, 95%CI = 79.9–99.9%, *p* = 0.031).

In the per-location (Table 3; II and III) analysis with a co-detection (positive read) on all modalities, a sensitivity of 80% (95%CI = 56.3–97.3%) was found for the combination of DWI + ultrafast-DCE + ^18^F-FDG-PET/CT, for DWI + DCE, and for PET + DWI. In this analysis, the specificity for the combination of DWI + ultrafast-DCE + ^18^F-FDG-PET/CT (specificity=97.4%, 95%CI = 93.5–99.3%) was non-significantly higher than DWI + DCE (specificity = 96.1% (95%CI = 91.7–98.6%, *p* = 0.157) and DWI + PET (specificity = 96.1%, 95%CI = 91.7–98.6%, *p* = 0.157).

**Figure 1:**
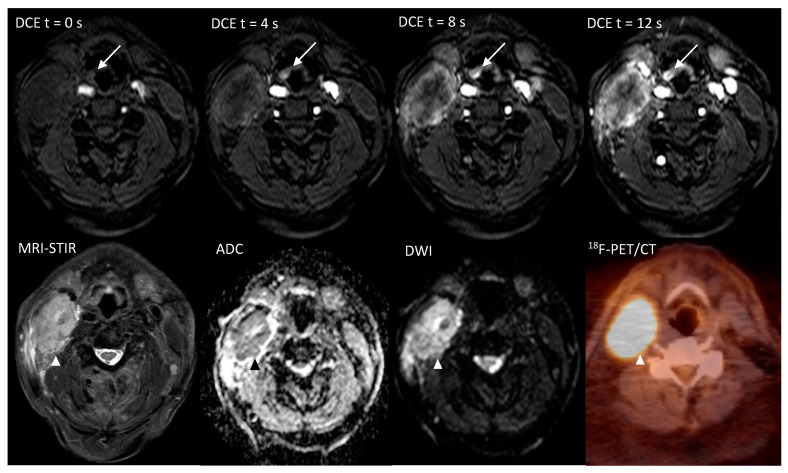
Imaging of a 71-year-old patient, with a hypopharyngeal (epiglottal) primary tumor detected only on the ultrafast-DCE imaging (arrow), whereas DWI and ^18^F-FDG-PET/CT did not detect the lesion. The overt malignant lymph node metastasis is marked with an arrowhead (Δ).

The per-patient analysis of (Table 4; II and III) with an independent positive read in either one of the combined modalities, resulted in a sensitivity of 100% for either DWI, ultrafast-DCE or PET and for either DWI or PET. In this analysis, an 11.1% (95%CI = 0.3–48.3%, *p* = 1.0) specificity was found for either DWI or DCE, either DWI or PET and for either DWI, DCE or PET. In one patient (5%), ultrafast-DCE provided decisive score when DWI and PET were scored as benign (Table 2; patient 19; Figure 1). In two patients (10%) ultrafast-DCE provided additional confidence to DWI for scoring probably malign lesion as malign (patient 5, 24).

In the per-patient (Table 4; II and III) analysis with a co-detection (positive read) on all combined modalities, a 95% (95%CI = 75.1–99.9%) sensitivity for DWI + PET, was non-significantly higher than DWI + DCE (sensitivity = 90% (95%CI = 68.3–98.8%, *p* = 0.32) and DWI + DCE + PET (sensitivity = 90% (95%CI = 68.3–98.8%, *p* = 1.0). In this analysis, the specificity of 55.6% (95%CI = 21.2–86.3) for DWI + DCE + PET was higher than DWI + ultrafast-DCE (specificity = 44.4%, 95%CI = 13.7–78.8%, *p* = 0.63) and DWI + ^18^F-FDG-PET/CT (specificity = 33.3%, 95%CI = 7.5–70.1%, *p* = 1.0).

### 2.3. Interobserver Agreement

The overall agreement in the per-location analysis was 87.9% for DWI, 86.8% for DCE, and 86.8% for ^18^F-FDG-PET/CT. In the per-patient analysis the overall agreement was 41.4% for DWI, 41.4% for DCE and showed a higher trend of 65.5% for ^18^F-FDG-PET/CT (Table 5). The specific positive agreement in the per-location analysis 76.6% for DWI, 77.6% for DCE, and 71% for ^18^F-FDG-PET/CT. In the per-patient analysis the specific positive agreement 82.8% for DWI, 82.6% for DCE, and 93.1% for ^18^F-FDG-PET/CT.

## 3. Discussion

Our results showed that high temporal resolution (ultrafast) DCE-MRI is feasible in the head and neck area and has the potential to improve the detection of clinically occult primary tumors in patients presenting with cervical SCC lymph node metastases. Qualitative reading of ultrafast-DCE images could depict faster signal enhancement compared with normal tissue. Combining ultrafast-DCE with DWI and ^18^F-FDG-PET/CT further improved the diagnostic accuracy of UPT detection and EUA, resulting in a 69% detection rate of UPT in our series.

**Figure 2:**
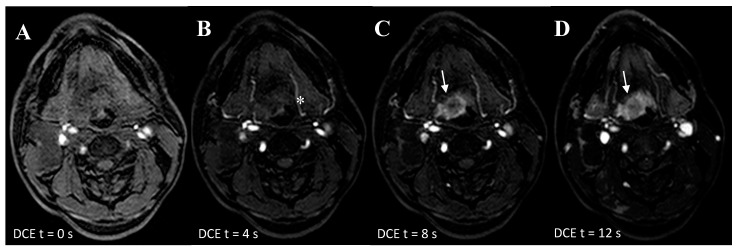
Ultra-fast DCE in a 53-year-old patient with an overt primary tumor in the tongue base. (**A**) At T = 0 seconds is the first frame in which the contrast enters the carotid and vertebral arteries. (**B**) At T = 4 seconds later, the contrast flows in the arterial branches of the external carotids (*). (**C**) At T = 8 seconds the contrast agent has ‘washed-in’ the hypervascular malignant lesion at the base of tongue (arrow). (**D**) At T =12 seconds, the contrast agent is still in the hypervascular lesion. At this stage, the surrounding oropharyngeal mucosa show also contrast enhancement (Δ).

The additional value of ultrafast-DCE over DWI and ^18^F-FDG-PET/CT is that it might capture complementary unique tumor characteristics. Although the precise physiological explanation of enhancement on DCE remains ambiguous (e.g., angiogenesis and increased microvessel density) [19], early intensity changes were indicative for malignancy (Figure 2). This is in line with studies in breast cancer with an earlier time of arrival of contrast bolus in the malignant lesion [15,16,17]. Although the thin mucosal hypervascular layer and normal tonsillar tissue might interfere with the detection UPT, no other hypervascular lesions are generally present in the head and neck area. This could provide additional value for UPT detection by adding detection based on lesion vascularity, to DWI as a sign of tissue cellular density and ^18^F-FDG-PET/CT as a sign of tumor metabolism. Due to the limited spatial resolution of ^18^F-FDG-PET/CT for detecting small primary lesions, the addition of ultrafast-DCE may provide more confidence for the radiologist to mark a suspicious lesion as being malignant or benign (Figure 1, Figures S2 and S3) or when DWI and ^18^F-FDG-PET/CT are inconclusive. Furthermore, a similar high, positive agreement was found for ultrafast-DCE (77.6%) compared to DWI (76.6%) and ^18^F-FDG-PET/CT (71%), although more experience is gained with DWI and ^18^F-FDG-PET/CT. Also, the addition of ultrafast-DCE might enhance demarcation of tumoral boundaries, in order to optimize radiotherapy.

In order to find all malignant lesions and consequently increase the efficiency of radiotherapy [6,21], a high sensitivity was considered being more important for guiding the otolaryngologist towards the potential primary tumor location rather than a high specificity. False positive findings as a result of a low to intermediate specificity of the diagnostic tests may result in unnecessary biopsies during the EUA, which outweighs the risk of missing an occult primary tumor. However, in clinical practice, in order to enhance treatment accuracy and reduce morbidity [6,21] the per-patient diagnostic accuracy of imaging modalities is more clinically relevant. This is because treatment is based on the whole patient, instead of based on one location. Therefore, enhancement of the per-patient diagnostic accuracy implies an enhanced detection of relevant lesions. The current study showed that co-detection on DWI, DCE and PET increased the per-patient specificity, whereas the sensitivity decrease was limited, compared with the co-detection on DWI and PET (Table 4).

Previously, Noij et al. [1] hypothesized that based on the different properties of the imaging modalities, a combination might improve the diagnostic accuracy. They found a sensitivity of 93.3% for a positive read on either ^18^F-FDG-PET/CT or DWI [1]. This study confirmed that hypothesis, and showed that the diagnostic accuracy could be enhanced further by adding ultrafast-DCE, resulting in the highest sensitivity (95%) when a positive read was found of a location on either ultrafast-DCE, DWI or ^18^F-FDG-PET/CT. This was in contrast with Godeny et al. [4], who found no improved diagnostic accuracy using contrast-enhanced T1-weighted fat-suppression combined with DWI (88.2% sensitivity). However, the authors did not evaluate the ultrafast-DCE modality nor assessed the accuracy of all modalities combined. Furthermore, the current prospective study showed a per-patient sensitivity of 100% for ^18^F-FDG-PET/CT compared with 95% for DWI and 90% for ultrafast-DCE. This was found to be higher than the reported per-patient sensitivity of 93.8% for ^18^F-FDG-PET/CT and 81.3% for DWI in Noij et al. [1], and 94.4% for ^18^F-FDG-PET/CT and 82.4% for DWI in Godeny et al. in [4], in relative small heterogeneous patient samples of these retrospective studies.

A second step in the diagnostic workup with respect to the high sensitivity, is to improve lesion specificity in order to reduce the amount of biopsies during the EUA, without compromising on the UPT the detection level. However, due to physiological processes or modality constraints, small lesions with low FDG-uptake in challenging sites (i.e., tonsils) are missed easily using only FDG-PET [4]. Similarly, the low ADC value of non-pathological tonsil-tissue on DWI could diminish detection accuracy. In the current study, we confirmed our hypothesis that the combination of modalities (ultrafast-DCE, DWI and ^18^F-FDG-PET/CT) could filter the suspect locations most accurate (97.4% specificity). In the per-patient analysis the combination of ultrafast-DCE, DWI and ^18^F-FDG-PET/CT yielded the highest specificity (55.6%). This was in contrast with Noij et al. [1], in which a high specificity for DWI and ^18^F-FDG-PET/CT (96.1% and 94.7%, respectively) was found; however, no improvement of diagnostic accuracy was found combining DWI with ^18^F-FDG-PET/CT (60%). Furthermore, to our knowledge, the use of ultrafast-DCE, combined with other modalities was not described previously in head and neck UPT.

Factors for the selection of the most optimal imaging modalities are the reproducibility of each modality, cost-effectiveness (i.e., ^18^F-FDG-PET/CT is more expensive), availability (i.e., DCE/DWI is more accessible than ^18^F-FDG-PET/CT and ultrafast-DCE could easily be acquired when MRI combined with DWI is already performed), additional information (e.g., ^18^F-FDG-PET/CT can provide whole-body information on the presence of distant metastases and (unknown) primary tumor outside the head and neck area).Reproducibility is dependent on the interobserver agreement, which was found to be excellent for all modalities in the per-location and moderate to good in the per-patient analysis.

Currently, ^18^F-FDG-PET/CT is the preferred imaging technique to perform in the work-up of UP HNSCC. However, an alternative reading approach might increase accuracy; by firstly selecting potential positive locations on either ^18^F-FDG-PET/CT, DWI or ultrafast-DCE, which would result in the highest detection accuracy. Thereafter, by selecting suspicious lesions with positive score for all modalities, the detection accuracy would have decreased (e.g., patient 19, 27 and 29 (Table 2)). However, some suspicious lesions at previous steps, had a primary tumor at different site, which underlines the need for further evaluation of suspicious patients by histopathological evaluation or optimizing imaging modalities.

Quantification based on delineated region of interest, has been introduced previously in DCE [15,16] in order to optimize detecting occult primary tumors. Although voluminous quantitative parameters did not showed increased accuracy [1], semi-quantitative parameters describing the area under the curve, time-to-peak or upslope angle might increase detection performance, and should be assessed in future studies.

In 14 of the 29 patients the occult primary tumor was located in the tonsils or base of tongue. Although in patients cervical lymph node metastasis of UPT during EUA tonsillectomy is routinely performed and mucosectomy of the base of tongue is increasingly applied to find the occult primary tumor, one could argue that almost half of the occult primary tumors would be detected regardless of imaging. However, if (combined) imaging is highly accurate half of these patients would be spared tonsillectomy and/or mucosectomy with their associated morbidity. Besides, in 17 (59%) patients the lymph node metastases were HPV-positive guiding the search for UPT to the oropharynx, focusing irradiation to the oropharynx and decreasing morbidity of extensive irradiation areas. In the present study the observers were blinded to the HPV-status to assess the diagnostic values of (combined) imaging techniques. However, in clinical practice HPV-status is likely to be available at the multidisciplinary meeting, but not always before EUA with tonsillectomy and eventual mucosectomy.

This study had some limitations. Ultrafast-DCE is not yet commonly used in the head and neck area, and due to the fact that UPTs are relatively rare, only a small patient population could be investigated. The discovered trend of higher diagnostic accuracy in the current study was not found significant, and should be validated by adding ultrafast-DCE to DWI-MRI and PET in larger patient groups. Secondly, both radiologists had no previous experience in using DCE-MRI for tumoral detection, therefore a learning curve may have led to an underestimation of the detection accuracy. Knowledge of the side of lymphadenopathy could have biased observations of the potential primary tumor site towards the ipsilateral side. However, in clinical practice this information was also known. Furthermore, in some patients all imaging modalities pointed towards one specific location for the UPT, however the EUA with extensive biopsies did eventually not confirm the HNSCC (Figure S3). This might be due to sampling errors with biopsies during EUA, due to which occult primary tumors could have been missed. Nowadays, the transoral robotic surgery performing mucosectomy of the base of tongue, have increased accuracy, which might provide a better reference standard in future studies.

## 4. Materials and Methods

For this prospective study HNSCC patients were consecutively included between July 2014 and February 2019 in our tertiary referral center. The ethical board waived the need for a written informed consent (Amsterdam UMC Medisch Ethische ToetsingCommissie (METC), reference: 2013.191). Inclusion criteria were: a pathologically proven cervical SCC nodal metastasis with a clinically occult primary tumor after clinical evaluation with flexible endoscopic examination, prior both to the examination of the upper digestive tract under general anesthesia (e.g., diagnostic endoscopy/biopsies); MR imaging (including ultrafast-DCE-MRI and DWI), and ^18^F-FDG-PET/CT. The imaging acquisitions were performed on the same day. Exclusion criteria were a prior history of HNSCC or malignancy requiring systemic or surgical treatment in the head and neck area. The reference standard for analysis was the final diagnosis, as given by a multi-disciplinary head and neck oncology team that reviewed all diagnostic modalities including imaging and biopsies.

### 4.1. Image Acquisition

MRI was performed on a 3T Achieva MR system (Philips Healthcare, Best, The Netherlands) with a 16-channel neurovascular coil. Axial T1-weighted (T1w), axial T2-weighted (T2w) and axial STIR images were obtained.

DWI (9 b-values: 0, 25, 50, 75, 150, 300, 500, 750 and 1000 s/mm^2^) was obtained using fat-suppressed single-shot spin-echo echo-planar imaging (SS-SE-EPI) with following parameters: TR 500 ms; TE 105 ms; echo-planar imaging factor 35; slice thickness 2 mm; intersection gap 0.3 mm; sensitivity encoding factor 3.5; field of view (FOV) 230 × 230 mm; 128 × 128 matrix. The ADC-map was produced using software provided by the manufacturer.

Ultrafast-DCE images were acquired using a T1-weighted fast-field-echo (FFE) sequence with the following parameters: TR 3.09 ms; TE 1.48 ms; flip angle 12; FOV 240 × 240 × 114 mm; slice-thickness 4.4 mm, 144 × 144 matrix; 75 dynamic acquisitions of 4s; signal averages 2. After three native acquisitions, an intravenous bolus injection of 0.2 mmol/kg Gd-DOTA (Dotarem, Guerbet, France) was administered (3 mL/s by power injector followed by 25 mL saline flush).

^18^F-FDG-PET/low-dose-CT was acquired according to the EANM 2.0 guidelines with an EARL-accredited Ingenuity TF or Gemini-TF PET/CT system (Philips Healthcare, Best, The Netherlands) [22]. The examination was performed after 6 h fasting and adequate hydration. Scans were acquired with arms down, from mid-thigh to skull vertex, 60 min after intravenous injection of ^18^F-FDG (2.5 MBq/kg, 3 min per bed position). Iterative ordered subsets expectation maximization was used to reconstruct the ^18^F-FDG-PET/CT images, using optimized head and neck area parameters (4 iterations, 16 subsets, 5 mm 3-dimensional Gaussian filter) and photon attenuation correction. Image matrix size was 144 × 144 with 4 × 4 × 4 mm voxel size. Low-dose-CT was acquired using 50 mAs, 120 kV for anatomical correlation and attenuation correction, using a 512 × 512 matrix size, resulting in 1.17 × 1.17 mm pixel size with a 5 mm slice thickness.

### 4.2. Qualitative Image Analysis

The observers were asked to find the primary tumor site of clinically occult HNSCC patients by evaluating one imaging modality retrospectively with knowledge of the lymph node metastasis location and were blinded to HPV-status, EUA findings and final diagnosis. A 3-point-scale was used: (1) benign, (2) probably malignant, (3) malignant. A high sensitivity to find the primary tumor location was considered to be more important than a high specificity. Therefore, a sensitive read of these scoring systems was used, which considered the neutral evaluation (probably malignant; score 2) also indicative of malignancy.

MR images were assessed by two radiologists (P.d.G., J.A.C.), with 13 and 33 years of experience with head and neck radiology, respectively. During the first reading session conventional MRI sequences (T1w, T2w, STIR) were allowed to be used combined with DWI. Asymmetrical lesions with a higher signal intensity on DWI (high b1000) and a low ADC were assigned as being potentially malignant. A second MR reading session after a 2 months’ time-interval (to limit recall bias) was performed using conventional MRI sequences combined with ultrafast-DCE. Moreover, a very early contrast enhancement on ultrafast-DCE (Figure 1) and distortion of normal anatomy were considered signs of malignancy.

The ^18^F-FDG-PET/CT images were assessed independently by two nuclear medicine physicians (G.J.Z., E.F.C.) with 6 and 25 years of experience with head and neck nuclear medicine, respectively. Furthermore, consensus scorings of DWI, DCE and ^18^F-FDG-PET/CT were used for final analysis.

### 4.3. Statistical Analysis

Two analyses were performed: (1) Per-location; systematic analysis of six separate locations: tonsil (left, right), base of tongue (BOT) (left, right), hypopharynx, other). (2) Per-patient; patients were scored according to the highest rated lesion. EUA with eventual biopsies was used as reference standard.

Outcomes were considered true positive if the biopsy, performed during EUA, was positive for malignancy combined with a positive read on imaging for the same location. When the biopsy was negative for malignancy and the imaging read was negative for malignancy, then results were considered true negative. A positive read on imaging combined with a biopsy negative for malignancy was considered false positive. In case the biopsy was positive for malignancy, but the lesion was not detected on imaging, findings were considered false negative.

Diagnostic accuracy (sensitivity and specificity) was measured for each scoring system, for each modality, and tested using the McNemar test. The overall agreement was calculated, which measures the observer variation in terms of probability of the other raters obtaining the same score. Furthermore, a proportion specific agreement was calculated to assess the agreement separately in positive ((probably) malignant lesions) and negative ratings ((probably) benign lesions) [23]. An interobserver agreement of 0.2–0.4 is stated as fair, 0.4–0.6; moderate, 0.6–0.8; good, above 0.8; excellent [1]. All statistic tests were performed with SPSS Statistics for Windows, version 22 (IBM Corp., Armonk, NY, USA).

## 5. Conclusions

Ultrafast-DCE showed potential to improve the detection of unknown primary tumors in addition to DWI and ^18^F-FDG-PET/CT in patients presenting with cervical squamous cell carcinoma lymph node metastasis. The combined use of modalities acquired highest sensitivity. The addition of ultrafast-DCE might yield reader confidence for suspicious locations.

## Figures and Tables

**Table 1:** Baseline characteristics.

	Clinically Occult Primaries *n* = 29 (%)
Age, median, range	60, 45–76
Gender, *n* (%)	
Male	20 (69)
Female	9 (31)
Primary tumor location, *n* (%)	
Tonsil	9 (31)
Base of tongue	3 (10)
Hypopharynx	5 (17)
Other	3 (10)
Retromolar trigone	1 (3)
Piriform sinus/Epiglottis	2 (7)
Unknown	9 (31)
T-stage **, *n* (%)	
Tx	9 (31)
T1	13 (45)
T2	5 (17)
T3	1 (3)
T4 *	1 (3)
N-stage **, *n* (%)	
N1	6 (21)
N2a	6 (21)
N2b	11 (38)
N2c	4 (14)
N3	2 (7)
AJCC-Stage, *n* (%)	
III	5 (17)
IV	24 (83)
HPV, *n* (%)	
Positive	17 (59)
Negative	9 (31)
Unknown	3 (10)

* This T4 primary remained occult in the clinic; ** 7th edition of AJCC staging.

**Table 2:** The final diagnosis per-patient (T-stage or unknown primary (UP)), tumor location, consensus scores of each modality for primary tumor location and false positive scores. A +++—score was rated for a malign lesion, a +—score for a probably malign lesion and a -—score for a benign lesion.

	Final	PT location	DWI		DCE		PET	
Patient			PT Location Score	False Positive	PT Location Score	False Positive	PT Location Score	False Positive
1	UP		-			+ Left Tonsil		+++ Left Tonsil
2	T1	Right Tonsil	+++		+++		+++	
3	T1	Left Tonsil	+++		+++		+++	
4	T1	Left Tonsil	+		+		+	
5	T1	Right Tonsil	+		+++		+++	
6	T3	Right Epiglottis/Piriform sinus	-		+		+++	
7	T2	Right base of tongue	+		+		+++	
8	UP		-		-		-	
9	UP		-	+ Left Tonsil	-		-	+ Left Tonsil
10	UP		-	+ Right Tonsil	-	+ Right Tonsil	-	+++ Right Tonsil
11	UP		-	+ Right Tonsil	-	+ Right Tonsil	-	+ Right Tonsil
12	UP			+ Left Tonsil	-		-	+ Left Tonsil + Right tonsil + Left Hypopharynx
13	T1	Left Hypopharynx	+		+		+	
14	T1	Right piriform sinus	+++		+		+++	+ Right Tonsil
15	T1	Right base of tongue	+++		+		+++	
16	UP			+ Right Tonsil		+ Right Tonsil	-	
17	T1	Right Tonsil	+++		+++		+++	
18	T1	Left Tonsil	+++		+++		+++	
19	T2	Left Hypopharynx	-	+ Right Tonsil	+	+ Right Tonsil	-	+ Left Tonsil + Right Tonsil
20	T1	Left Tonsil	+		+	+ Right Tonsil	+	+ Right Tonsil
21	T1	Right Tonsil	+		+		+	
22	T1	Left Hypopharynx	+++		+++		+++	
23	T1	Left Hypopharynx	+		+		+++	
24	T1	Right base of tongue	+		+++		+++	
25	UP			+ Left Hypopharynx		+ Left Tonsil + Left Hypopharynx		+++ Left Tonsil
26	UP			+ Left Tonsil		+ Left Hypopharynx		+++ Left Hypopharynx
27	T1	Left hypopharynx	-	+ Left Tonsil	-		+	
28	T4	Left Retromolar trigone	+++		+++		+++	
29	T2	Left Tonsil	-		-		-	+++ Right base of tongue

**Table 3:** The per-location diagnostic accuracy of (I) single use of DWI, DCE or ^18^F-FDG-PET/CT and (II) the combined use of DWI + DCE and separately PET + DWI; of which the first line comprised a co-detection on both imaging modalities rated positively for malignancy. The second line comprised an independent detection on DWI or DCE and separately PET + DWI, in which either one was rated positively. (III) The combination of DWI + DCE + PET; in which the first line comprised a co-detection on all DWI + DCE + PET rated positively for malignancy. The second line comprised an independent detection on DWI, DCE or PET, in which either one was rated positive.

	Per-Location (*n* = 174)	AUC (95%CI)	Sensitivity (%, 95%CI, Ratio)	Specificity (%, 95%CI, Ratio)	YI
I	DWI	0.92 (0.87–0.96)	80 (56.3–94.3, 16/20)	93.5 (88.4–96.8, 144/154)	0.735
	DCE	0.93 (0.88–0.96)	90 (68.3–98.8, 18/20)	92.9 (87.6–96.4, 143/154)	0.829
	^18^F-FDG-PET/CT	0.90 (84.1–93.8)	90 (68.3–98.8, 18/20)	89.6 (83.7–94, 138/154)	0.796
II	Co-detection on DWI and DCE	0.94 (0.90–0.97)	80 (56.3–97.3, 16/20)	96.1 (91.7–98.6, 148/154)	0.761
	Either DWI or DCE	0.90 (0.84–0.94)	90 (68.3–98.8, 18/20)	90.3 (84.4–94.5, 139/154)	0.803
	Co-detection on PET + DWI	0.94 (0.90–0.97)	80 (56.3–94.3, 16/20)	96.1 (91.7–98.6, 148/154)	0.761
	Either PET or DWI	0.87 (0.81–0.92)	90 (68.3–98.8, 18/20)	87 (80.7–91.9, 134/154)	0.770
III	Co-detection on DWI and DCE and PET	0.95 (0.91–0.98)	80 (56.3–94.3, 16/20)	97.4 (93.5–99.3, 150/154)	0.774
	Either DWI or DCE or PET	0.87 (0.81–0.92)	95 (75.1–99.9, 19/20)	86.4 (79.9–99.9, 133/154)	0.814

Abbreviations: AUC = area under the curve, YI = Youden Index, 95%CI = 95% Confidence interval. PET = ^18^F-FDG-PET/CT.

**Table 4:** The per-patient diagnostic accuracy of I) single use of DWI, DCE or ^18^F-FDG-PET/CT. II) The combined use of DWI + DCE and separately PET + DWI; of which the first line comprised a co-detection on both imaging modalities rated positively for malignancy. The second line comprised an independent detection on DWI or DCE and separately PET + DWI, in which either one was rated positively. III) The combination of DWI + DCE + PET; in which the first line comprised a co-detection on all DWI + DCE + PET rated positively for malignancy. The second line comprised an independent detection on DWI, DCE or PET, in which either one was rated positive.

	Per-Patient (*n* = 29)	AUC (95%CI)	Sensitivity (%, 95%CI, Ratio)	Specificity (%, 95%CI, Ratio)	YI
I	DWI	0.72 (0.53–0.87)	95 (75.1–99.9, 19/20)	22.2 (2.8–60, 2/9)	0.117
	DCE	0.72 (0.53–0.87)	90 (68.3–98.8, 18/20)	33.3 (7.5–70.1, 3/9)	0.233
	^18^F-FDG-PET/CT	0.76 (0.56–0.90)	100 (100, 20/20)	22.2 (2.8–60, 2/9)	0.222
II	Co-detection on DWI and DCE	0.76 (0.56–0.90)	90 (68.3–98.8, 18/20)	44.4 (13.7–78.8, 4/9)	0.344
	Either DWI or DCE	0.69 (0.49–0.85)	95 (75.1–99.9, 19/20)	11.1 (0.3–48.3, 1/9)	0.061
	Co-detection on DWI and PET	0.76 (0.56–0.90)	95 (75.1–99.9, 19/20)	33.3 (7.5–70.1, 3/9)	0.283
	Either DWI or PET	0.72 (0.53–0.87)	100 (100, 20/20)	11.1 (0.3–48.3, 1/9)	0.111
III	Co-detection on DWI and DCE and PET	0.79 (60.3–92.0)	90 (68.3–98.8, 18/20)	55.6 (21.2–86.3, 5/9)	0.456
	Either DWI or DCE or PET	0.72 (52.8–87.3)	100 (100, 20/20)	11.1 (0.3–48.3, 1/9)	0.111

Abbreviations: AUC = area under the curve, YI = Youden Index, 95%CI = 95% Confidence interval. PET = 18F-FDG-PET/CT.

**Table 5:** The overall and specific agreement of DWI, DCE and PET per-location and per-patient, using a cut-off between 1 versus ≥ 2 on the 3-point scale as a malignant lesion.

Per-Location		DWI			DCE			^18^F-FDG-PET/CT		
		Total	*n*	% (95%CI)	Total	*n*	% (95%CI)	Total	*n*	% (95%CI)
Overall Agreement	Mean	174	153	87.9 (82.1–92.4)	174	151	86.8 (80.8–91.4)	174	151	86.8 (80.8–91.4)
Specific Agreement	Total	174	163	93.7 (89–96.8)	174	163	93.7 (89–96.8)	174	156	89.7 (84.1–93.8)
	Positive	23.5	18	76.6 (54.8–91.4)	24.5	19	77.6 (56.4–91.8)	31	22	71 (52–85.8)
	Negative	150.5	145	96.3 (92–98.7)	149.5	144	96.3 (91.9–98.7)	143	134	93.7 (88.4–97.1)
**Per-patient**										
Overall Agreement	Mean	29	12	41.4 (23.5–61.1)	29	12	41.4 (23.5–61.1)	29	19	65.5 (45.7–82.1)
Specific Agreement	Total	29	24	82.8 (64.2–94.2)	29	21	72.4 (52.8–87.3)	29	27	93.1 (77.2–99.2)
	Positive	23.5	21	89.4 (69.8–98.1)	23	19	82.6 (61.8–98.1)	27	26	96.3 (81–99.9)
	Negative	5.5	3	55 (13.2–91.5)	6	2	55 (13.2–91.5)	2	1	50 (1.26–98.7)

Abbreviations: 95%CI = 95% Confidence interval.

## Supplementary Materials

The following are available online at https://www.mdpi.com/2072-6694/12/10/2826/s1, Figure S1: Patient inclusion; Figure S2: Tumor detection with ultrafast-DCE only; Figure S3: False positive lesion detection on all modalities; Table S1: Distribution of positive patients and lesions among the imaging modalities.
