# Supplementary material for: The Additional Value of Ultrafast DCE-MRI to DWI-MRI and 18F-FDG-PET to Detect Occult Primary Head and Neck Squamous Cell Carcinoma
Source: Cancers (Basel). 2020 Sep 30;12(10):2826. doi: 10.3390/cancers12102826 (PMC7600235; doi:10.3390/cancers12102826)
Supplement: Supplementary file 1 [file cancers-12-02826-s001.pdf]

Supplementary Materials

The Additional Value of Ultrafast DCE-MRI to DWI-MRI and 18F-FDG-PET to Detect Occult Primary Head and Neck Squamous Cell Carcinoma

Roland M. Martens, Ruud van der Stappen, Thomas Koopman, Daniel P. Noij, Emile F. Comans, Gerben J. Zwezerijnen, Marije R. Vergeer, C. René Leemans, Remco de Bree, Ronald Boellaard, Jonas A. Castelijns and Pim de Graaf

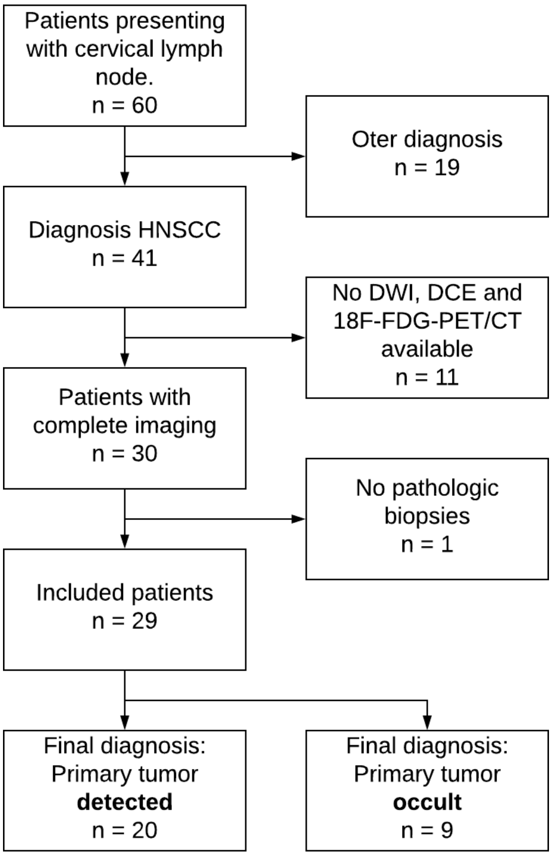

Figure S1. Patient inclusion.

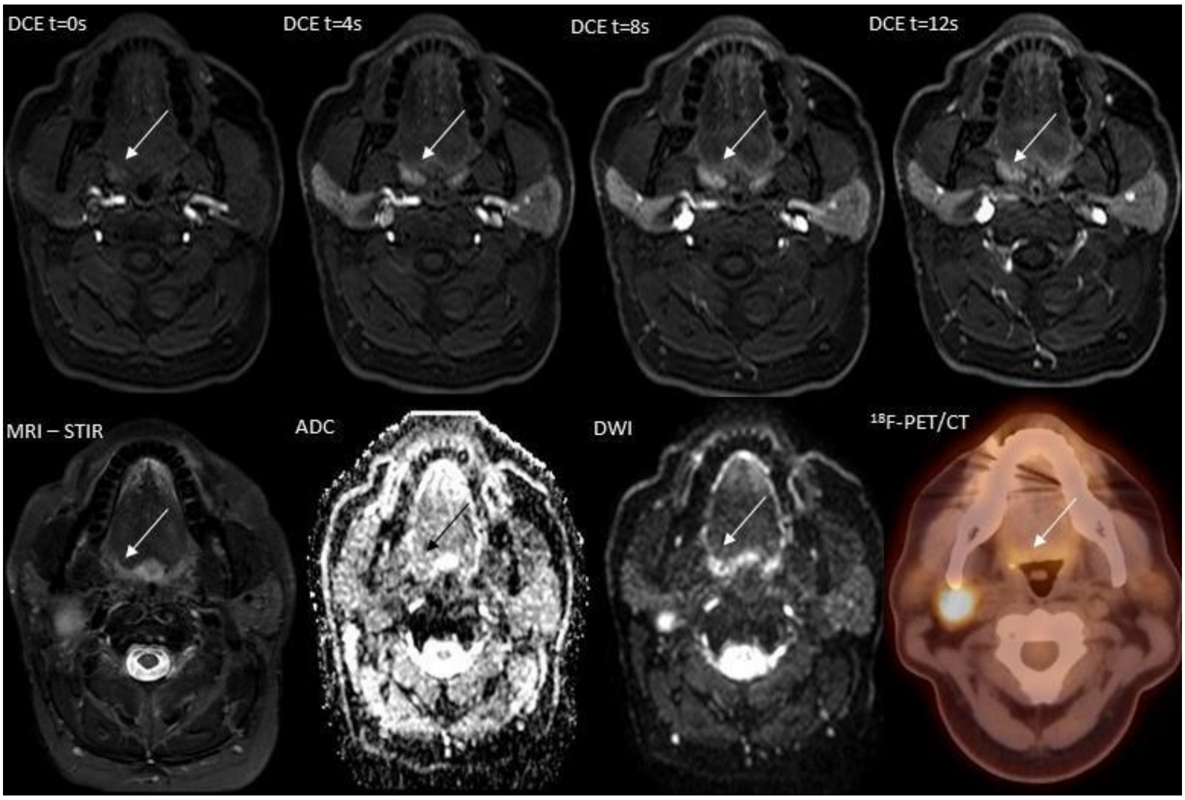

Figure S2. Tumor detection with ultrafast-DCE only**.** Imaging of a 57-year-old male patient diagnosed with a T1N1 HNSCC in the right tonsil (HPV positive). DCE is shown throughout time (4 seconds between frames). A lesion is detectable asymmetrical signal intensity, which was found suspect for malignancy. No suspect lesions were detected on 18F-FDG-PET/CT or DWI.

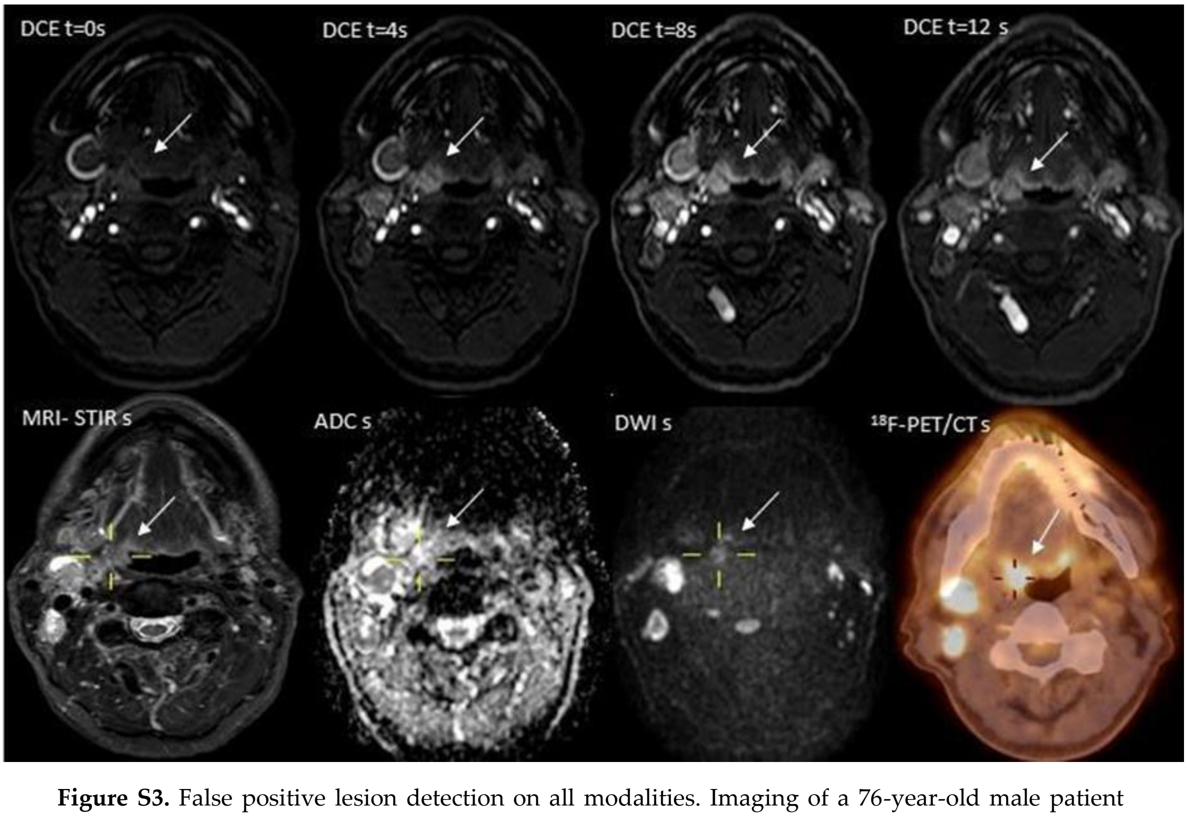

Figure S3. False positive lesion detection on all modalities. Imaging of a 76-year-old male patient diagnosed with a histological proven T0N2b (HPV-negative). On all image modalities a possible suspect lesion was scored as false-positive by each observer.

Table S1. Distribution of positive patients and lesions among the imaging modalities.

	Per-patient analysis				Per-location analysis				
	**Total**	**DCE**	**DWI**	**^{18}F-FDG-PET/CT**	**Total**	**DCE**	**DWI**	**^{18}F-FDG-PET/CT**	**EUA**
Primary tumor location, n (%)	29 (100)				40 (100)				20
Tonsil	8 (27.6)	8	8	8	23 (57.5)	16	17	20	8
Base of tongue	6 (20.7)	6	6	6	11 (27.5)	8	6	10	6
Hypopharynx	1 (3.4)	1	1	1	1 (2.5)	1	1	1	1
Other location	5 (17.2)	4	5	5	5 (12.5)	4	2	3	5
Unknown	9 (31.0)	6	7	7	-	-	-	-	-
T-stage*, n (%)	29 (100)				40 (100)				20
Tx	9 (31.0)	6	7	7	21 (52.5)	11	10	16	0
T1	13 (44.8)	12	13	13	13 (32.5)	12	12	13	13
T2	5 (17.2)	4	4	5	5 (12.5)	3	3	3	5
T3	1 (3.4)	1	1	1	1 (2.5)	1	0	1	1
T4	1 (3.4)	1	1	1	1 (2.5)	1	1	1	1

The distribution per-patient and per-lesion, who were scored positively for malignancy, among the primary tumor location and T-stage. In the per-patient analysis most primary tumors were detected by all imaging modalities. However in the Tx category, the EUA did not confirm malignancy. In the per-lesion analysis, there were more lesions in the tonsil, base of the tongue area and Tx stage which were scored malignant on the imaging modalities, but they were also not confirmed by the EUA.

© 2020 by the authors. Licensee MDPI, Basel, Switzerland. This article is an open access article distributed under the terms and conditions of the Creative Commons Attribution (CC BY) license (http://creativecommons.org/licenses/by/4.0/).
